# Supplementary material for: Age-Related Sex Differences in Glucose Tolerance by 75 g Oral Glucose Tolerance Test in Japanese
Source: Nutrients. 2022 Nov 17;14(22):4868. doi: 10.3390/nu14224868 (PMC9698682; doi:10.3390/nu14224868)
Supplement: Supplementary file 1 [file nutrients-14-04868-s001.zip › nutrients-1997480-supplementary.pdf]

Supplementary

Materials and Methods

1.1. Classification of Study Subjects in the Results of 75g Oral Glucose Tolerance Test

As shown in Figure 1B, we classified subjects into the diabetes group (PG0 >= 7.0 mmol/L or PG120 >= 11.1 mmol/L), combined glucose intolerance (CGI) group (7.0 mmol/L > PG0 >= 6.1 mmol/L and 11.1 mmol/L > PG120 >= 7.8 mmol/L), the IGT group (PG0 < 6.1 mmol/L and 11.1 mmol/L > PG120 >= 7.8 mmol/L), IFG group (7.0 mmol/L > PG0 >= 6.1 mmol/L and PG120 < 7.8 mmol/L), high-normal fasting plasma glucose (high-normal FPG) group (6.1 mmol/L > PG0 >=5.6 mmol/L and PG120 < 7.8 mmol/L), and NGT (PG0 < 5.6 mmol/L and PG120 < 7.8 mmol/L) group according to Japanese Clinical Practice Guideline for Diabetes 2019 [1] (Figure 1B).

1.2. Calculation Formula for Indicators of Insulin Secretion and Insulin Resistance

We also calculated the homeostasis model assessment of insulin resistance (HOMA-IR, fasting plasma glucose [FPG (= PG0)] (mg/dL) x IRI0 (μU/mL)/405), β cell function (HOMA-β, IRI0 (μU/mL) x 360/[PG0 (mg/dL) − 63]) [2], and Matsuda index of insulin sensitivity (10,000/square root of [fasting glucose (mg/dL) x fasting insulin (μU/mL)] x [mean glucose (mg/dL) x mean insulin (μU/mL) during 75gOGTT]), as reported [3]. We calculated the insulinogenic index by dividing the increment in serum insulin (μU/mL) by the increment in plasma glucose (mg/dL) during the 0-30 min time periods of the 75gOGTT [(IRI30 (μU/mL) – IRI0 (μU/mL)) / (PG30 (mg/dL) − PG0 (mg/dL))] [4]. The insulin secretion/insulin resistance (disposition) index was calculated as insulinogenic index x Matsuda index [5].

References

1. Araki, E.; Atsushi Goto, A.; Kondo, T.; Noda, M.; Noto, H.; Origasa, H.; Osawa, H.; Taguchi, A.; Tanizawa, Y.; Tobe, K.; Yoshioka, N. Japanese Clinical Practice Guideline for Diabetes 2019. *Diabetol Int*. **2020**. 11, 165-223. doi: 10.1007/s13340-020-00439-5.
2. Matthews, D.R.; Hosker, J.P.; Rudenski, A.S.; Naylor, B.A.; Treacher, D.F.; Turner, R.C. Homeostasis model assessment: insulin resistance and beta-cell function from fasting plasma glucose and insulin concentrations in man. *Diabetologia*. **1985**. 28, 412-419.
3. Matsuda, M. Insulin sensitivity indices obtained from oral glucose tolerance testing: comparison with the euglycemic insulin clamp. *Diabetes Care*. **1999**. 22, 1462-1470.
4. Kosaka, K.; Kuzuya, T.; Yoshinaga, H.; Hagura, R.; A prospective study of health check examinees for the development of non-insulin-dependent diabetes mellitus: relationship of the incidence of diabetes with the initial insulinogenic index and degree of obesity. *Diabet Med*. **1996**. 13, S120-126.
5. Weiss, R.; Taksali, S.E.; Tamborlane, W.V.; Burger, T.S.; Savoye, M.; Caprio, S. Predictors of Changes in Glucose Tolerance Status in Obese Youth. *Diabetes Care*. **2005**. 28, 902-909.

Table S1. Spearman's correlation analyses between glycemic parameters and lipid parameters among participants divided into young males, young females, elderly males, and elderly females.

		insulinogenic index		HOMA-β		HOMA-IR		Matsuda Index		disposition Index	
		ρ	*p*	*ρ*	*p*	*ρ*	*p*	*ρ*	*p*	*ρ*	*p*
BMI	young males	0.078	0.079	0.239	<0.01*	0.251	<0.01*	-0.223	<0.01*	-0.084	0.061
	young females	0.031	0.608	0.022	0.716	0.079	0.190	-0.024	0.692	0.017	0.781
	elderly males	0.124	0.163	0.282	<0.01*	0.364	<0.01*	-0.315	<0.01*	-0.010	0.913
	elderly females	0.074	0.248	0.395	<0.01*	0.512	<0.01*	-0.515	<0.01*	-0.233	<0.01*
TG	young males	0.088	0.049*	0.259	<0.01*	0.300	<0.01*	-0.311	<0.01*	-0.152	<0.01*
	young females	0.104	0.084	0.183	<0.01*	0.073	0.223	-0.113	0.059	0.075	0.212
	elderly males	0.286	<0.01*	0.287	<0.01*	0.259	<0.01*	-0.248	<0.01*	0.170	0.055
	elderly females	0.119	0.064	0.282	<0.01*	0.235	<0.01*	-0.227	<0.01*	-0.035	0.584
HDL-C	young males	-0.115	0.010*	-0.190	<0.01*	-0.221	<0.01*	0.262	<0.01*	0.101	0.024*
	young females	0.066	0.275	-0.057	0.346	-0.054	0.367	0.041	0.494	0.107	0.076
	elderly males	-0.185	0.037*	-0.317	<0.01*	-0.260	<0.01*	0.256	<0.01*	-0.073	0.417
	elderly females	-0.081	0.208	-0.175	<0.01*	-0.242	<0.01*	0.213	<0.01*	0.070	0.278
LDL-C	young males	0.099	0.026*	0.237	<0.01*	0.238	<0.01*	-0.282	<0.01*	-0.121	<0.01*
	young females	-0.042	0.487	0.033	0.584	0.022	0.718	-0.081	0.176	-0.057	0.348
	elderly males	-0.029	0.748	-0.018	0.838	0.062	0.488	-0.044	0.624	-0.060	0.502
	elderly females	-0.003	0.966	0.013	0.845	0.170	<0.01*	-0.146	0.023*	-0.061	0.339
TG /HDL-C	young males	0.100	0.025*	0.275	<0.01*	0.322	<0.01*	-0.345	<0.01*	-0.171	<0.01*
	young females	0.073	0.230	0.193	<0.01*	0.079	0.187	-0.118	0.050	0.030	0.622
	elderly males	0.269	<0.01*	0.341	<0.01*	0.314	<0.01*	-0.311	<0.01*	0.127	0.153
	elderly females	0.122	0.057	0.274	<0.01*	0.272	<0.01*	-0.258	<0.01*	-0.053	0.410
LDL-C /HDL-C	young males	0.138	<0.01*	0.272	<0.01*	0.291	<0.01*	-0.347	<0.01*	-0.135	<0.01*
	young females	-0.072	0.230	0.061	0.310	0.047	0.439	-0.089	0.137	-0.108	0.074
	elderly males	0.117	0.190	0.228	0.010*	0.229	0.010*	-0.209	0.018*	0.012	0.895
	elderly females	0.046	0.469	0.131	0.040*	0.272	<0.01*	-0.232	<0.01*	-0.085	0.184

BMI, body mass index; TG, triglyceride; HDL-C, high density lipoprotein-cholesterol; LDL-C, low density lipoprotein-cholesterol; HOMA-IR, homeostasis model assessment insulin resistance; HOMA-β, homeostasis model assessment beta cell. *, indicated significant difference ($p < 0.05$).
